# From Host Heme To Iron: The Expanding Spectrum of Heme Degrading Enzymes Used by Pathogenic Bacteria

**DOI:** 10.3389/fcimb.2018.00198

**Published:** 2018-06-19

**Authors:** Kristin V. Lyles, Zehava Eichenbaum

**Affiliations:** Biology, Georgia State University, Atlanta, GA, United States

**Keywords:** pathogenic bacteria, heme oxygenase, heme degradation, heme binding, iron regulation

## Abstract

Iron is an essential nutrient for many bacteria. Since the metal is highly sequestered in host tissues, bound predominantly to heme, pathogenic bacteria often take advantage of heme uptake and degradation mechanisms to acquire iron during infection. The most common mechanism of releasing iron from heme is through oxidative degradation by heme oxygenases (HOs). In addition, an increasing number of proteins that belong to two distinct structural families have been implicated in aerobic heme catabolism. Finally, an enzyme that degrades heme anaerobically was recently uncovered, further expanding the mechanisms for bacterial heme degradation. In this analysis, we cover the spectrum and recent advances in heme degradation by infectious bacteria. We briefly explain heme oxidation by the two groups of recognized HOs to ground readers before focusing on two new types of proteins that are reported to be involved in utilization of heme iron. We discuss the structure and enzymatic function of proteins representing these groups, their biological context, and how they are regulated to provide a more complete look at their cellular role.

## Introduction

In the late 1960s, Tenhunen and colleagues isolated the first heme oxygenase, HO-1 (Tenhunen et al., 1968, 1969). While the enzyme was discovered in rats, HO-1 is highly conserved in mammals. The discovery of HO-1 provided valuable insights into the pathway that reduces hemoglobin to bile (Ostrow et al., 1962). Previous to this discovery, research showed that heme is cleaved to generate the linear tetrapyrrole biliverdin, which is subsequently reduced to bilirubin by biliverdin reductase. Yet, the mechanism by which heme was converted to biliverdin was not understood. Almost 30 years after discovering HO-1, the first prokaryotic HO, HmuO, was described in the Gram-positive Corynebacterium (Schmitt, 1997b; Wilks and Schmitt, 1998). Schmitt and colleagues screened a genomic library of *Corynebacterium diphtheriae* in *Corynebacterium ulcerans* mutants that were unable to grow in medium containing heme or hemoglobin as iron sources. The clone selected based on its ability to restore growth carried a gene (*hmuO*) with high homology to HO-1. Biochemical characterization revealed that, in the presence of a redox partner, HmuO catalyzes *in vitro* the oxidative cleavage of heme to produce biliverdin IXα (Wilks and Schmitt, 1998; Kunkle and Schmitt, 2007). Two more bacterial HOs, HO-1 and HemO, were then cloned from *Synechocystis* spp. *PCC 6803* of the Cyanobacteria phylum, and the Gram-negative Proteobacterium, *Neisseria meningitides* respectively (Cornejo et al., 1998; Zhu et al., 2000). Both HOs also share overall structural and mechanical similarities with the mammalian HO-1. The discovery of bacterial enzymes that are comparable to the mammalian counterparts promoted the initial notion that heme degradation represents an evolutionarily conserved mechanism. The mechanism these bacterial and mammalian HOs use to cleave the heme ring is referred to as “canonical heme oxidation” (Wilks and Heinzl, 2014). The discovery of IsdG and IsdI, two paralogous HOs from *Staphylococcus aureus*, and MhuD, a similar enzyme from *Mycobacterium tuberculosis*, gave rise to the second family of HOs due to their unique structure and novel mechanism for cleaving the protoporphyrin ring (Skaar et al., 2004a; Chim et al., 2010; Nambu et al., 2013). These enzymes demonstrated that oxidative heme catabolism could be achieved by more than one class of bacterial enzymes.

Since the discovery of HmuO, heme degradation in bacteria has become an active research area. Currently HOs are divided into the canonical HOs, which share structure and mechanistic similarities to HO-1, and the IsdG-like group of HOs (Wilks and Heinzl, 2014). Many potential HOs have also been identified based on their ability to carry out aerobic heme degradation *in vitro*. However, the acceptance of these proteins as HOs has met with some skepticism over the reaction mechanism and whether these proteins catalyze ring opening *in vivo* (Wilks and Ikeda-Saito, 2014). Non-enzymatic degradation of heme to verdoheme can be achieved by the addition of hydrogen peroxide (H_2_O_2_) to the solution (known as coupled oxidation). Coupled oxidation can take place in the presence of molecular oxygen (O_2_) and a reducing agent that converts the heme iron to ferrous iron (Fe^+2^) and H_2_O_2_. Various hemoproteins (e.g., myoglobin) and cytochrome mutants, in which the axial ligand was removed or replaced with a weaker ligand, readily exhibit this reaction (Avila et al., 2003). While oxidative cleavage by a HO or breakdown by coupled oxidation is achieved by two separate mechanisms, they cannot be discriminated based on their products alone as they both yield CO and verdoheme (which is hydrolyzed to biliverdin under acidic or alkali conditions). Because of this complexity and since proteins that exhibit heme degradation *in vitro* (in the presence of a reductant) may have another function under physiological conditions, it was suggested that a comprehensive approach that combines genetics, mechanistic enzymology, and metabolite profiling is necessary for the characterization these proteins before terming them HOs (Wilks and Ikeda-Saito, 2014). Such extensive studies are not yet available for all cases but in this review, we attempt to compel what information exists. We briefly explain the HO-1 and the IsdG-like HOs before focusing on two new types of bacterial proteins that are reported to be involved in iron gain from heme. We discuss the structure and enzymatic function of these proteins, the biological context, and regulation mode. For this paper, we reviewed the published literature as well as structural information. Protein structures were generated by downloading the Protein Data Bank file into PyMol (Table 1) and sequence comparisons were generated through NIH Protein BLAST.

**Table 1:** Proteins and their corresponding Protein Data Bank Identity (PDB) used for bioinformatical analysis.

**Protein**	**PDB**
HO-1	1N3U
HmuO	3I8R
PigA	1SK7
HemO	1P3T
IsdG	1XBW
MhuD	4NL5
HmoB	4FVC
Isd-LmHde	4KIA
ChuS	4CDP
PhuS	4MF9
HemS	2J0P
HugZ	3GAS
HutZ	3TGV
HupZ	5ESC
ChuZ	3SWJ

Lastly, many bacteria degrade heme to obtain the iron. Here we focus on infectious bacteria, where heme catabolism is particularly important since it allows pathogens to leverage on the host heme pools. Nevertheless, it is important to note that non-pathogenic bacteria also use HOs and that these enzymes can be involved in additional functions. For example, in the photosynthetic Cyanobacteria, HO-1 catalyzes the first step in the production of phycocyanobilin, the tetrapyrrole chromophore the bacteria use to harvest solar energy (Cornejo et al., 1998).

## Heme is a key iron source for pathogenic bacteria within the host environment

Although iron is a common element on Earth, very little of it is free in host tissues, partially because iron is insoluble under physiological conditions (Sánchez et al., 2017). Additionally, free ferric ions can interact with H_2_O_2_ through the Haber-Weiss reaction to generate free radicals (Valko et al., 2005). This can lead to cell death due to oxidative stress, which injures tissues through lipid peroxidation and can damage genetic material. Sequestration of iron is vital for the host because iron is an essential nutrient for most invading microorganisms. Iron overload is linked to increase in both the quantity of incidents and the severity of bacterial infections in patients as can be seen in hemodialysis patients (Ishida and Johansen, 2014). Evidences linking iron availability to increased susceptibility were also obtained in murine infection models with multiple bacterial pathogens including *Vibrio vulnificus, Listeria monocytogenes, Yersinia pestis, and Yersinia pseudotuberculosis* (Sword, 1966; Quenee et al., 2012; Arezes et al., 2015; Miller et al., 2016). For these reasons, the host iron is tightly sequestered in proteins.

Most mammalian iron is bound to heme which is predominately in complex with hemoglobin (67% of the total iron) but also found in myoglobin and cytochromes (3.5 and 3% of the total iron, respectively). A significant but smaller fraction of the body iron (25%) is stored in the cellular compartment in ferritin or hemosiderin. The plasma contains only 0.1% of the iron in the body, where it is mobilized as ferric ion bound to transferrin, a glycoprotein that is maintained under physiological conditions only at 30–40% saturation level. Hemoglobin and heme that are liberated by lysis are captured by the plasma proteins haptoglobin and hemopexin, respectively. The resulting complexes are then cleared by macrophages. Ferric iron is cleared from secretions (e.g., tears, saliva, etc.) by lactoferrin. Therefore, under physiological conditions the majority of a host's iron is bound to proteins and held in the intracellular compartment. In response to infection, the innate immune system orchestrates a systemic reduction in the iron levels and bioavailability by inhibiting the dietary uptake of iron and its export into circulation, increasing the level of heme- and ferric- binding proteins in the serum, and releasing apo-lactoferrin into the infection site (Johnson and Wessling-Resnick, 2012; Ganz and Nemeth, 2015; Knutson, 2017).

As a result, pathogenic bacteria have developed mechanisms that compete for the host's iron during infection (Carver, 2018). A significant fraction of the strategies used by bacteria to obtain iron from the host is dedicated to heme acquisition (Sheldon et al., 2016; Huang and Wilks, 2017). Many bacteria secrete hemolysins that can rupture erythrocytes and other blood cell types. Some intracellular pathogens, such as *Salmonella*, activate hemophagocytic macrophages to take up erythrocytes (Silva-Herzog and Detweiler, 2008). Heme is not only readily taken up and used by numerous bacterial pathogens, it serves as the preferred iron source for important pathogens such as S. *aureus* and *Streptococcus pyogenes* (Eichenbaum et al., 1996; Skaar et al., 2004b). Bacteria employ soluble and/or surface proteins that can seize heme from hemoproteins (e.g., hemoglobin, hemopexin etc.). Heme is then shuttled across the outer-membrane and/or the cell wall onto dedicated transporters for import across the cytoplasmic membrane. Inside the bacterial cell, heme is incorporated directly into proteins or it is degraded to release the iron (Sheldon and Heinrichs, 2015). Oxidative degradation by HOs is the most common mechanism used to liberate iron from heme, although other mechanisms have been discovered.

## The structure and function of heme degrading enzymes

### Canonical heme oxygenases

The first discovered group of HOs are usually referred to as canonical HOs, and include mammalian-HO-1, HmuO, and PigA/HemO. They are α-only proteins and typically contain nine to ten helixes. Their catalytic sites are highly conserved, especially the proximal α-helix (**Figure 2A**) (Hirotsu et al., 2004). Comparative studies between apo- and holo-HO-1 and HmuO show that heme binding induces a conformational change where the proximal α-helix twists in on itself, tightening the helix and shortening it. This results in the proximal α-helix moving toward the heme molecule. Concurrently, the distal α-helix swings toward the heme, causing the proximal and distal α-helix to open and close around the heme-binding pocket.

**Figure 1:**
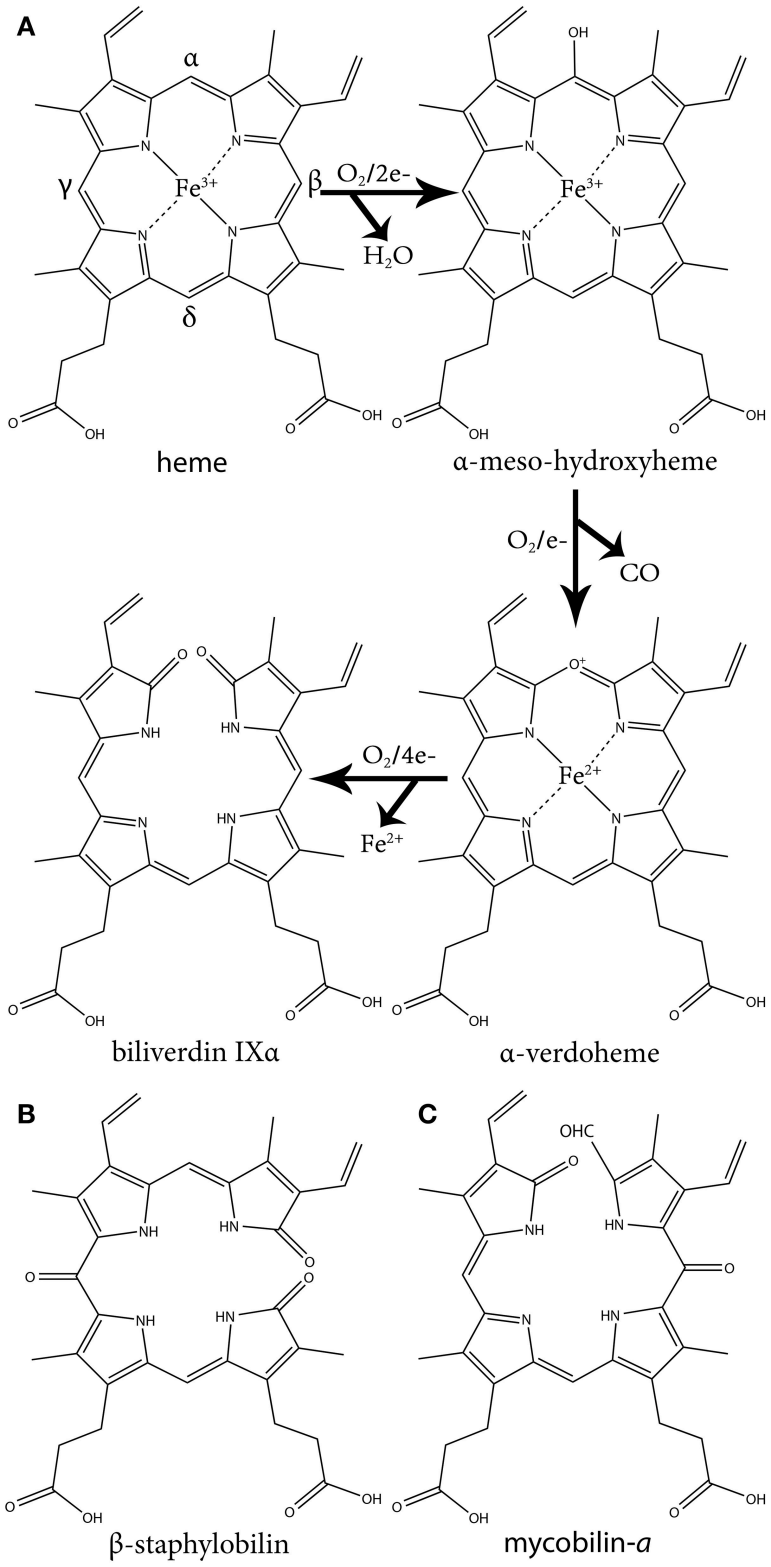
The three oxidative steps of canonical heme oxidation and the chemical structure of staphylobilin and mycobilin. **(A)** The three oxidative steps of canonical heme oxidation. In the first step heme is oxidized to ferric-hydroperoxide, then it is self-hydroxylated to α-meso-hydroxyheme. Next it is oxidized to α-verdoheme, which is ultimately oxidized to α-biliverdin. **(B)** One of the two isomers of staphylobilin produced by IsdG/I. **(C)** One of the two isomers of mycobilin produced by MhuD.

This group of HOs degrade heme to α-biliverdin (a linear tetrapyrrole), CO and free ferrous iron (Fe^+2^) by three successive oxygenation steps and consumes three O_2_ and seven electrons (Figure 1A). This mechanism has been extensively reviewed in several articles (Wilks and Heinzl, 2014; Wilks and Ikeda-Saito, 2014). Briefly, the reaction is initiated by the reduction of the heme ferric iron (Fe^+3^) and O_2_ binding. The coordinated oxygen (Fe^+2^-O_2_) is then reduced to peroxide (Fe^+3^-OOH), a reactive species that hydroxylates the heme on the α-meso carbon. In the second step, the α-meso-hydroxyheme is oxidized to α-verdoheme while releasing CO. In the last step, the verdoheme ring is cleaved, generating Fe^+3^-biliverdin complex that is reduced to release the iron (Fe^+2^). This differs from coupled oxidation in the first step, were peroxide from the solvent (H_2_O_2_) instead of coordinated peroxide (Fe^+3^-OOH) reacts with the heme iron to form the meso-hydroxyheme (which in turn progress to yield verdoheme). Hence, unlike HO catalysis, coupled oxidation is inhibited by the H_2_O_2_ scavenging enzyme, catalase.

**Figure 2:**
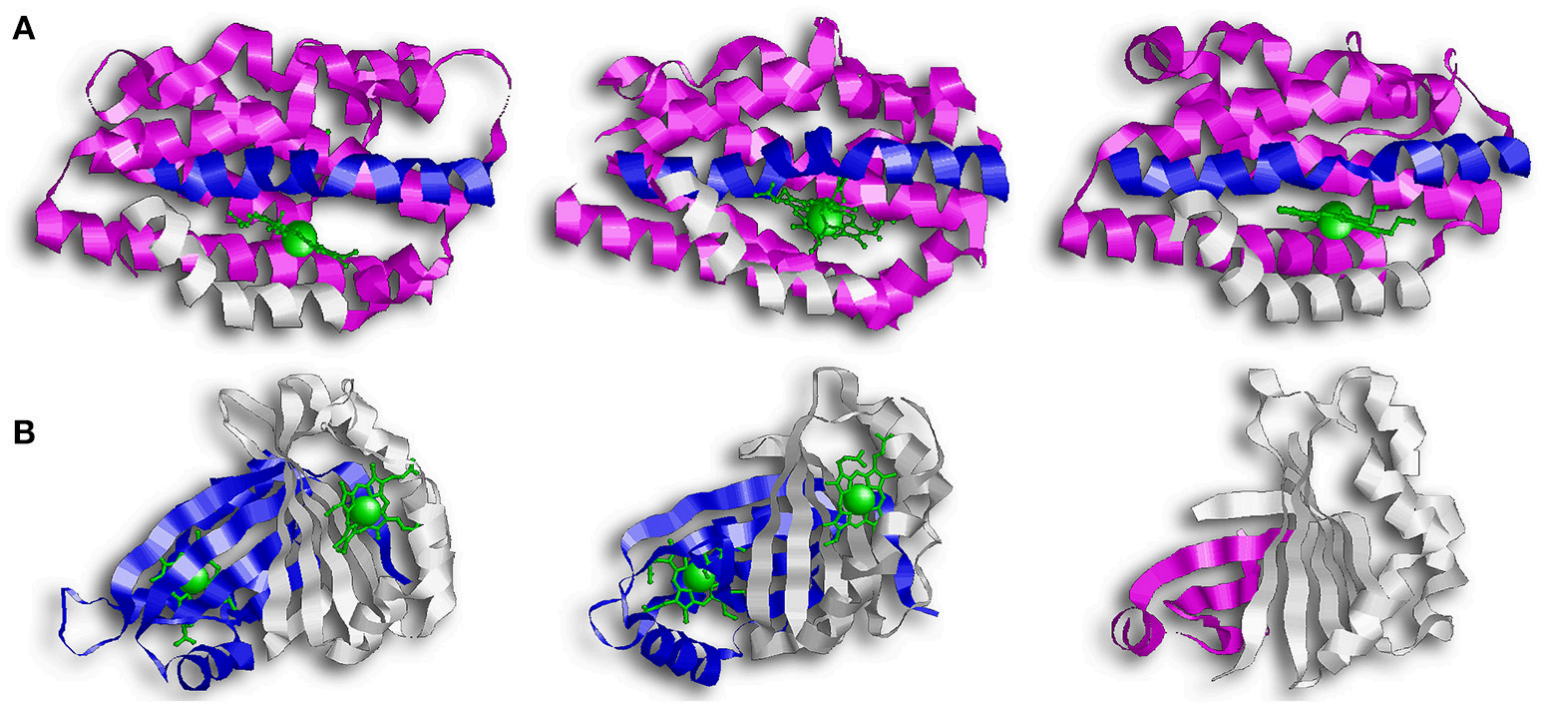
Overall structure of canonical and IsdG-like heme oxygenases. **(A)** From left to right: HO-1, HmuO, and PigA. This family of enzymes are commonly referred to as the canonical HOs. They are α-only proteins and have been colored so that the proximal helix is in white and the distal helix is in blue. These helixes take an open conformation when the binding pocket is empty but tighten and close around the heme molecule. Note how the propionate groups in PigA are rotated compared to HO-1 and HmuO. **(B)** From left to right: IsdG, MhuD, Isd-LmHde. These enzymes represent the second group of HOs, the IsdG-like HOs. This group consists of α/β proteins that dimerize across their β-sheets. Both IsdG and MhuD have been colored so that one monomer is in white and the second monomer is in blue. The Isd-LmHde structure has been colored so that the N-terminal is in magenta and the C-terminal is in white.

Regioselectivity is the preference of a specific chemical bond over other possible bonds during a reaction. For this group of HOs the regioselectivity is for the α-meso carbon over the β-, γ-, or δ-meso carbons. Regioselectivity is controlled during the hydroxylation step due to steric hindrance of the distal helix interacting with the protoporphyrin ring and allowing ring opening only at the α-meso carbon (Figure 2A) (Unno et al., 2013). The distal helix also bends, bringing the reactive iron dioxygen species closer to the α-meso carbon. The interactions between the distal helix and the protoporphyrin ring are stabilized through an extensive hydrogen bond network that is supplied by water molecules. This network also serves as the proton relay network required for oxygen activation (Unno et al., 2007, 2013).

While the regioselectivity of the α-meso carbon is a defining characteristic of this group of bacterial HOs, a notable exception exists in PigA/HemO. Interestingly, PigA/HemO produces a mixed product of β- and δ-biliverdin in a 3:7 ratio (Friedman et al., 2004). A structure study of heme bound PigA/HemO shows that the heme is rotated about 100° in the binding pocket as compared to HO-1 and HmuO (Figure 2A). This heme rotation is believed to result in the favoring of the δ-meso carbon over the α-meso carbon in PigA/HemO during the hydroxylation step (Friedman et al., 2004; Fujii et al., 2004). Mutagenic analysis of HO-1 and HmuO show that mutations to the amino acids that interact directly with the propionates or through solvent interactions are important for proper placement of the heme in the binding site (Unno et al., 2007). In PigA/HemO, substituting Lys34 and/or Lys132 with alanine residues allows the production of α-biliverdin by the mutant enzymes in addition to β- and δ-biliverdin (Fujii et al., 2004). It was suggested that these residues interact with the propionate of the rotated heme molecule and that when the interactions are lost, it releases the ring rotation allowing the positioning the of α-meso carbon at the oxidation site. In addition, the ratio of β- and δ-biliverdin in PigA/HemO is greatly influenced by the amino acid identity at position 189, which is occupied by phenylalanine. A F189W mutant was found to produce mostly β-biliverdin (~90%) during heme catalysis. These observations suggest that steric interactions bewteen the amino acid residue at position 189 with the heme impact the enzyme's topology and hence the reaction regiospecifificy.

### The IsdG family of heme oxygenases

The second group of bacterial HOs are commonly referred to as both non-canonical and IsdG-like HOs (Wilks and Heinzl, 2014). The founder of this group, IsdG, and its paralog, IsdI (64% sequence identity and 79% sequence similarity), were discovered in *S. aureus*, and degrade heme to staphylobilin and formaldehyde instead of biliverdin and CO (Figure 1B) (Skaar et al., 2004a; Reniere et al., 2010; Matsui et al., 2013). Following the discovery of IsdG/I, MhuD was discovered in *M. tuberculosis*. During heme degradation, MhuD retains its meso-carbon as an aldehyde and generates mycobilin as its final product (Figure 1C) (Nambu et al., 2013). MhuD shares 24% sequence identity and 46% sequence similarity with IsdG. Both of these enzymes are α+β class of protein and are part of the dimeric α- and β-barrel superfamily (Figure 2B). MhuD and IsdG/I contain a ferredoxin-like fold, consisting of three α-helices and four β-strands arranged in a βαββααβ pattern, in their ABM domain (PF03992) (Figure 2B). These enzymes also have similar active site structures. In IsdG, enzymatic activity is supplied by the catalytic triad Asn7, Trp67, and His77 (Wu et al., 2005). His77 serves as the axial ligand, Trp67 induces heme ruffling, and Asn7, which is critical for catalytic activity, acts to stabilize the reaction intermediates and contributes to heme distortion. Mutagenesis of key residues in MhuD, showed that its heme ligand is a conserved histidine residue (His75) and that a mutation of Asn7 to alanine resulted in decreased enzymatic activity (Nambu et al., 2013).

Advancements in time-resolved *in proteo* mass spectrometry have allowed for the characterization of the heme-derived species formed during IsdG mediated heme degradation (Streit et al., 2016). Using this process, Streit and colleagues observed the production of an intermediate species with the same molecular weight as mycobilin (a formyloxobilin with α regiospecificity) (Figure 1C). Further analysis revealed this intermediate consists of mixed β/δ-formyloxobilin isomers which is consistent with the reported production of both β- and δ-staphylobilin by IsdG (Reniere et al., 2010). Overall, the data suggests that in the IsdG reaction, an initial rapid conversion of heme to meso-hydroxyheme is followed by the formation of formyloxobilin, which resolves into staphylobilin by releasing CH_2_O. More research is needed to understand why MhuD does not release formaldehyde from its formyloxobilin product. Interestingly, while a non-catalytic conversion of formyloxobilin to staphylobilin occurs when the product is extracted from the enzyme under aerobic conditions, *in proteo* MS analysis revealed that IsdG converts only a fraction of the produced formyloxobilins to staphylobilins (Streit et al., 2016). Hence, it seems possible that the remained formyloxobilin population might have a biological function.

**Figure 3:**
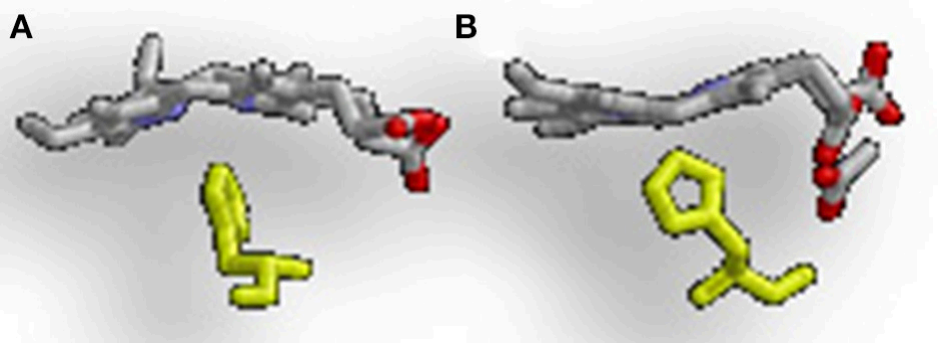
Alternative heme ruffling in IsdG and MhuD. **(A)** Represents the heme ruffling in IsdG and **(B)** Represents the heme ruffling in MhuD. Notice in **(A)**, how the IsdG bends the heme so that the β- and δ-meso carbons are pushed away from the histidine, causing the α-meso carbon to move toward the ligand. In **(B)**, MhuD bends the heme so that the β- and δ-meso carbons are closer to the histidine ligand.

The difference in regioselectivity of staphylobilin and mycobilin may be explained by a 90° in-plane rotation of the heme in the catalytic site which results in alternative forms of heme ruffling (Figure 3). Heme ruffling occurs when steric forces from the protein cause the usually planar heme molecule to bend. In IsdG/I, this results in the β- and δ-meso carbons being pushed toward the distal ligand (Lee et al., 2008; Takayama et al., 2015). This is distinct from the MhuD-induced heme ruffling, which leads to the α- and γ-meso carbons being exposed to reactive iron-dioxygen species (Graves et al., 2014). However, since the final product for MhuD are all α-formyloxobilins, other interactions must also be important for its regiospecificity. This differs from HmuO catalyzed heme oxidation, where water molecules associated with the HO push the α-meso carbon toward the reactive iron dioxygen species.

In addition to a putative *isdG* ortholog, the food-born pathogen *L. monocytogenes* codes for a second protein, Isd-LmHde, which shares structurally similarity with IsdG (Figure 2B) (Duong et al., 2014). Isd-LmHde is a monomer, and the full-length protein forms a β-barrel that is highly similar to the dimmers formed by IsdG/I and MhuD. The C-terminal region of Isd-LmHde shares 58% sequence similarity and 26% sequence identity with the IsdG monomer, but Isd-LmHde has an additional domain at the protein amino terminus. The N-terminal domain of Isd-LmHde consists of a pseudo-ferredoxin-like fold, arranged βαβββ, while the C-terminal domain of the enzyme has of the same ferredoxin-like fold found in the IsdG-like HOs (βαββααβ). Interestingly, an isolated C-terminal fragment of Isd-LmHde forms a homodimer in solution similar to the IsdG protein (Duong et al., 2014). Both the full-length protein and isolated C-terminal domain of Isd-LmHde degrade heme *in vitro* (in the presence of catalase, favoring enzymatic degradation). However, activity by the truncated enzyme is significantly reduced compared to the full-length protein, demonstrating that both protein regions in Isd-LmHde are necessary for full function.

### Enzymes with HemS motifs

This group consists of heme-binding and degrading proteins, intracellular heme shuttling proteins, and possibly have other functions. We discuss these proteins together due to overall structural similarity. The group's founder member, HemS, is associated with the heme-specific transport system in *Yersinia enterocolitica* (Stojiljkovic and Hantke, 1994). HemS alleviates heme toxicity in *Escherichia coli* and is required for *Y. enterocolitica* to utilize heme as an iron source (Stojiljkovic and Hantke, 1992; Schneider et al., 2006). It also contains tandem repeats of a domain termed the HemS motif (PF05171). This motif is also found in the heme shuttling protein PhuS of *Pseudomonas aeruginosa*, and in HmuS from the related *Y. pseudotuberculosis* and ChuS from *E. coli* O157:H7, both of which degrade heme *in vitro*.

**Figure 4:**
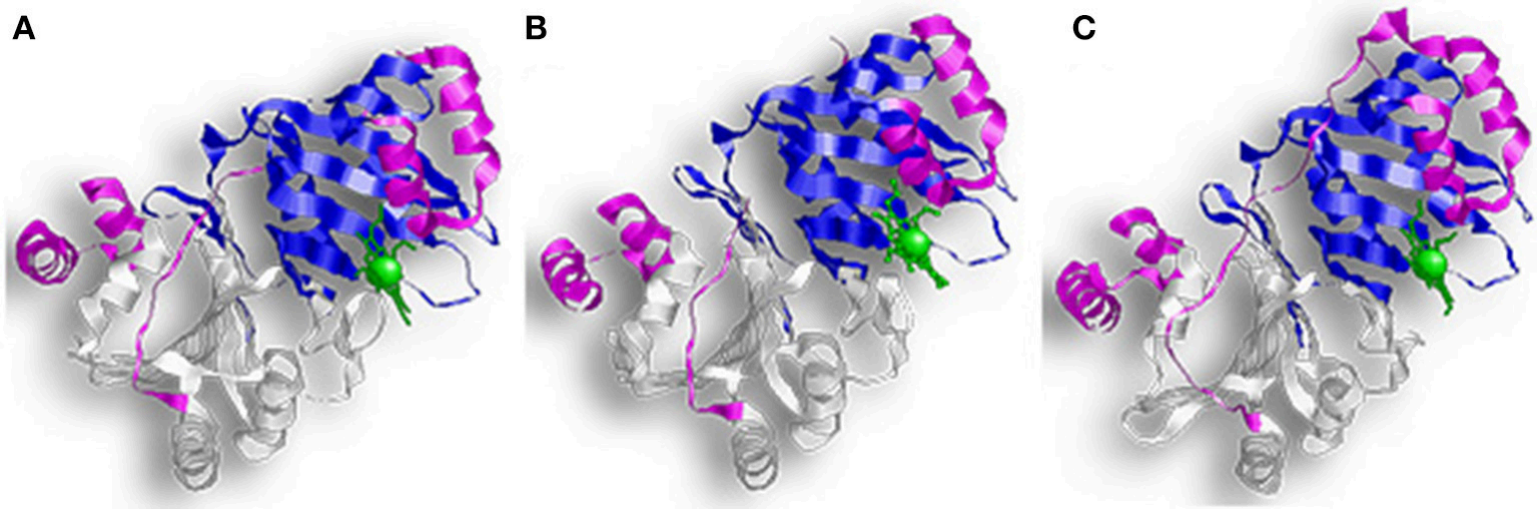
Overall structure of proteins with HemS motifs. **(A)** Represents ChuS, **(B)** Represents HemS, and **(C)** Represents PhuS. In all representations the N-terminal HemS domain is in white and the C-terminal HemS domain is in blue. Looking at the structure, ChuS consists of a mix of α-helices and β-strands arranged so that the β-sheets form the core of the enzyme and are flanked by three α-helices on one side and two on the other side. This configuration is conserved in the structure of HemS, although HemS is symmetrical, with three α-helices on each side.

HemS and ChuS function as monomers; while PhuS crystalized as a dimer, the monomeric form is more stable and the dominant form in solution (Figure 4) (Schneider and Paoli, 2005; Suits et al., 2006; Block et al., 2007; Tripathi et al., 2013). HemS shares 35% sequence identity and 51% similarity to PhuS, and 34% sequence identity and 49% similarity to ChuS. Looking at the structure, ChuS consists of 10 α-helices and 18 β-strands arranged so that two β-sheets of nine antiparallel β-strands form the core of the enzyme. The core is flanked asymmetrically by three α-helices arranged in an α-loop-α-loop-α motif on the C-terminal side and a pair of parallel α-helices on the N-terminal. This configuration is conserved in the structure of HemS, although HemS is symmetrical, with three α-helices arranged in an α-loop-α-loop-α on each side. For ChuS the carboxy and amino termini share limited primary sequence homology, but they represent structural duplication in its tertiary structure (Suits et al., 2005). The two halves appear to be associated across a central β-sheet, with key residues being conserved between both halves (i.e., Arg29/209, Phe127/304, and Tyr138/315). In the crystalized form, the PhuS dimers align across the β-sheets, forming a structure highly similar to ChuS. The C-terminal side of PhuS consists of three α-helices arranged in an α-loop-α-loop-α motif and a pair of parallel α-helices on the N-terminal side (Figure 4C).

The binding pocket in PhuS and HemS is more open than in ChuS, where the C-terminal α-helices are closer to the binding site (Schneider and Paoli, 2005; Lee et al., 2014). This supports studies showing that PhuS acts *in vivo* as an intracellular heme transport protein (Bhakta and Wilks, 2006). PhuS shuttles heme to PigA/HemO, a HO in *P. aeruginosa* (O'neill and Wilks, 2013). Upon binding heme, the C-terminal domain of PhuS rearranges to facilitate PhuS and PigA/HemO interactions, and heme transfer (Deredge et al., 2017). Heme-induced conformational changes were also reported for HemS, which alters between an apo-form that is open to a closed, heme-bound state (Schneider et al., 2006).

The heme binding residues for these proteins are located on their α7 helix with a conserved histidine acting as the iron axial ligand (His196 for HemS, His193 for ChuS, and His209 for PhuS) (Schneider and Paoli, 2005; Suits et al., 2006; Tripathi et al., 2013). Studies of a PhuS H209A mutant revealed that it can alternatively bind heme using His212 and that His210 helped stabilize these interactions (O'neill et al., 2012). Further analysis showed that the H209A mutant could still form a complex with the PigA/HemO, although to a less extend than the wild type protein. In comparison, while H212A and H210A mutants bound heme, they lost the ability to interact with PigA/HemO. Therefore, the current model suggests His209 and His212 of holo-PhuS act as a ligand switch for PigA/HemO (O'neill et al., 2012; Tripathi et al., 2013).

ChuS completes several cycles of aerobic heme degradation *in vitro* when either ascorbic acid or cytochrome P450 reductase (CPR)/NADPH are used as electron sources (Suits et al., 2005, 2006). Interestingly, both the N-terminal and the C-terminal halves of ChuS are tandem repeats that retained enzymatic activity, but the activity of the isolated domains varies depending on which reductant is used in the reaction (Suits et al., 2005). Catalase was found to inhibit this reaction in a concentration dependent manner, indicating that H_2_O_2_ is required for activity. While the exact mechanism is not fully understood, UV/Visual stop flow spectroscopy, NMR spectroscopy, and EPR analysis have provided insights into the reaction intermediates and products (Ouellet et al., 2016). Ouellet and colleges suggest that the first intermediate, α-meso hydroxyheme generated by H_2_O_2_ attack of ChuS-bound heme, is a short-lived intermediate that progresses to ferric verdoheme. A subsequent ring cleavage releases Fe^+3^, hematic acid (a monopyrrole moiety) and a tripyrrole product (containing both vinyl groups and one propionate). The tetrapyrrole ring cleavage in this reaction is different from simple hydrolysis of verdoheme, which produces a mixture of the four biliverdin isomers. This reaction also takes place in the presence of ascorbate or CPR/NADPH without externally added H_2_O_2_. The enzyme accelerates the production of H_2_O_2_ by ascorbate, suggesting that ChuS promotes H_2_O_2_-coupled heme degradation. The *chuS* gene is associated with heme-uptake machinery and is regulated due to iron availability, implying a function in supplying the bacteria with heme iron. The ability to release iron from heme under a relatively mild peroxide concentration, and the absence of known HOs in *E. coli* that degrade heme under aerobic conditions, are consistent with this proposal. Nevertheless, genetic analysis and addition biochemical characterization are required to test this hypothesis, and the possibility that ChuS serves alternative roles (e.g., protection from oxidative stress).

An ortholog of HemS from *Bartonella henselae's* was tested for heme degradation *in vivo* using the *E. coli* strain FB8.27 (Liu M. et al., 2012). This strain is engineered to take up heme, but it cannot release the iron from the porphyrin ring due to inactivation of the enzymes *efeB* and *yfeX*. Therefore, FB8.27 cannot grow using externally added heme as the sole source of iron. A recombinant *B. henselae* HemS protein restored growth of FB8.27 using heme iron, suggesting that HemS promotes the release of iron from heme *in vivo*. Currently the degradation products have not been characterized, and while it degrades heme in the presence of ascorbate or CPR/NADPH, HemS's sensitivity to catalase has not been investigated (Liu M. et al., 2012). Interestingly, a reduction of *hemS* gene expression does not impact growth, although it did increase the bacterial sensitivity to H_2_O_2_, implicating this enzyme in oxidative stress protection.

HmuS, a close HemS ortholog from *Y. pseudotuberculosis*, degrades heme to mixed biliverdin β- and δ-isomers *in vitro* (Onzuka et al., 2017). Heme degradation using either ascorbate or ferredoxin/NADPH both with and without catalase produced biliverdin while consuming molecular oxygen and releasing CO. The spectral readings of reactions performed with and without catalase remained the same. Although the reaction was slower with catalase, the heme was eventually converted to biliverdin. When HmuS was incubated with only H_2_O_2_, verdoheme accumulated as the final product. Heme degradation (but not binding) was abolished when the proposed axial ligand (His196) or a distal arginine (Arg102) was mutated to alanine. Together the data is consistent with enzymatic degradation of heme, but direct evidence that supports its function in heme catabolism *in vivo* is still missing.

In summary, evolution of the HemS family may reflect individual adaptation of different bacteria to their growth environment. While some proteins from this family (e.g., PhuS) serve in intracellular heme shuttling, others may contribute to protection from oxidative stress and/or iron supply (e.g., *B. henselae* HemS and ChuS). *In vitro*, many of the family members are reported to exhibit heme degradation activity, yet biliverdin was confirmed only with HmuS from *Y. pseudotuberculosis*. Non-enzymatic cleavage was not ruled out in all cases and at least with ChuS, heme cleavage by H_2_O_2_-driven reaction is suggested, albeit by a unique enzymatic mechanism that seems possible under physiological conditions. Hence more investigations, specifically with whole bacteria, are required to gain a better understanding of the function of proteins from this family.

### Enzymes with FMN-binding domains

The FMN binding-like superfamily is divided into the NADH FMN oxidoreductase-like subfamily and the pyridoxamine 5'-phosphate oxidase (PNPOx)-like subfamily (PF01243). Several proteins from the PNPOx-like subfamily bind and/or degrade heme *in vitro*. Based on this proliferation, Hu et al. (2011) recommend adding a third subfamily to the FMN superfamily that includes these heme-binding proteins (Hu et al., 2011). HutZ (*Vibrio cholerae*), ChuZ (*Campylobacter jejuni*), HugZ (*Helicobacter pylori*), and HupZ (*S. pyogenes*) are examples of heme-binding proteins with PNPOx-like domains that could belong to this third subfamily. While they all contain FMN-like binding domain, neither HupZ nor HutZ bound FMN *in vitro* (HupZ data not published) (Liu X. et al., 2012).

**Figure 5:**
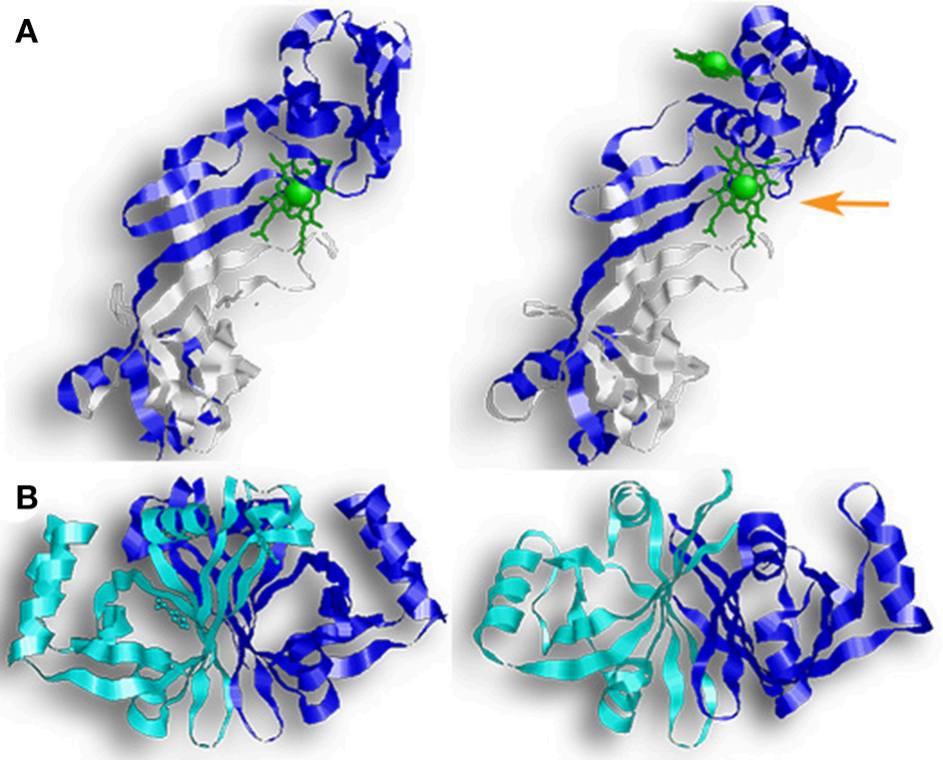
Enzymes with FMN-binding domains. Proteins from the PNPOx-like subfamily contain a β-barrel with a Greek key topology (a protein structure consisting of four antiparallel strands connected by three hairpin loops that is named for a common pattern in Grecian decoration). The pictured enzymes also contain FMN-binding domains, although neither HutZ or HupZ can bind FMN. **(A)** (from left to right) a monomer of HugZ and a monomer of ChuZ. In both representations, the PNPOx domain is in white. In ChuZ, the canonical heme binding pocket is indicated by the orange arrow. **(B)** (from left to right) HutZ and HupZ. In both representations one monomer is colored blue and the other monomer is colored teal.

All four of these enzymes crystallized as dimers, and size-exclusion assays for HugZ, ChuZ, and HutZ confirmed that they purify as dimers (Hu et al., 2011; Zhang et al., 2011; Uchida et al., 2012). Looking at their genetic sequence, the C-terminal regions are more conserved than the N-terminal region. HugZ and ChuZ have the highest level of similarity (54% sequence identity and 68% sequence similarity). Their N-terminal regions contain a simple α/β-type domain arranged in a three-stranded anti-parallel β-sheet, stacked against two α-helices in HugZ and three α-helices in ChuZ (Figure 5A). Their C-terminal regions contain the PNPOx-like domain and consist of eight anti-parallel β-stands and four α-helices. In ChuZ, the β-barrel takes on a distorted form with β10 and β11 extending out on one end of the barrel. HutZ and HupZ are smaller than HugZ and ChuZ, and share more similarity to the C-terminal region of HugZ and ChuZ than the N-terminal region (Figure 5B). In HutZ and HupZ, the antiparallel split-barrels are formed by six β-strands and helices α1 and α2 block the open ends of the β-barrel. HutZ has two more α-helices that are packed against the side of the β-barrel and HupZ, the smallest of all, has only one additional α-helix.

Dimerization in these proteins occurs across their β-sheets with one monomer packed at about 90° from the second monomer. Crystal structures of holo-HugZ and ChuZ show that the heme-binding pocket lies near the dimerized β-sheets and that heme binding is stabilized through interactions with residues from both monomers (Figure 5A). In ChuZ, the heme is bound between neighboring dimers using hydrophobic interactions between the protoporphyrin ring and the side-chains of His9 and His14 on different dimers (Zhang et al., 2011). The heme molecule binds with the propionic groups pointed toward the center of the dimer (Figure 5A). This is distinctive from canonical HOs where the propionic group is parallel to the enzyme (Figure 2A). Site directed mutagenesis of HugZ confirmed that His245 is involved in heme iron coordination and Arg166 interacts with the carboxylate group of the propionates (Hu et al., 2011). In ChuZ, His245 is also involved in heme iron coordination but Arg166 is not conserved and instead Lys166 interacts with the carboxylate group (Zhang et al., 2011). Currently only apo-crystal structures exist for HutZ and HupZ, but analyses using single site substitution mutagenesis of HutZ have shown that His63 and Arg92 (corresponding to His245 and Arg166 in HugZ) interact with the propionate due to shifts in heme-bound spectra (Liu X. et al., 2012). HupZ, however, lacks His245 and contains a glycine instead of Arg166, making it difficult to determine the heme coordinating residues based on sequence analysis alone (Sachla et al., 2016). Interestingly, ChuZ crystallized not only with the expected heme binding site, but with an additional surface bound heme molecule (Zhang et al., 2011). If ChuZ can also bind heme via the surface pocket *in vivo*, it may serve as a mechanism for preventing heme toxicity.

While all four proteins bind heme and mediate heme degradation *in vitro*, their physiological role is still under debate. A recent biophysical analysis of HutZ demonstrated that in the presence of exogenically added H_2_O_2_, heme is degraded to verdoheme (possibly β- or δ-isomer) and CO (Uchida et al., 2012). In the presence of ascorbate, the HutZ reaction is suggested to produce ferric biliverdin based on the appearance of broad featureless absorption in the visible region but this reaction is inhibited by catalase, which suggests coupled oxidation over enzymatic activity (Wyckoff et al., 2004). The *hutZ* gene is encoded by the iron-regulated *hutWXZ* operon that is divergently expressed from a heme import system in *V. cholera*. This operon compliments the growth of *hmuO* mutants in *Cyanobacteria* on heme iron, although complementation requires an intact *hutZ* gene (but not *hutW* or *X*). In addition, a *V. cholera* mutant that lost *hutZ* could not efficiently use heme as a source of iron. Since the *hutZ* mutant is not more sensitive to heme toxicity, it's phenotype is likely related to a defect in acquisition of heme iron (Wyckoff et al., 2004). *In vivo* studies implicate HutZ in heme catabolism, but more research is required.

*In vitro* degradation of heme by HugZ, ChuZ, and HupZ was observed in the presence of catalase (Ridley et al., 2006; Guo et al., 2008; Sachla et al., 2016). All three proteins degrade heme to produce CO and a chromophore that absorbs at 660 nm (in the presence of ascorbate or CPR/NADH). HPLC chromatography has confirmed that HugZ produce δ-biliverdin (Guo et al., 2008). While the chromophore produced by the ChuZ and HupZ reactions were not identified, the stability of HupZ's chromophore under acidic conditions suggests it is not verdoheme (Sachla et al., 2016). The iron-regulated expression of all three enzymes is consistent with a role in iron acquisition. Genetic studies are available only for *chuZ* where inactivation of this gene impaired the ability of *C. jejuni* to use heme or hemoglobin as a source of iron. Notably, the loss of *chuZ* had no impact on the bacterial sensitivity to oxidative stress (Ridley et al., 2006). In summary, similarly to the proteins with HemS domains, heme-binding proteins from the PNPOx-like subfamily might have evolved to play separate roles in different bacterial species, and more comprehensive investigations are required to describe the reaction catalyzed by these proteins and to determine their role in bacterial physiology.

## The genetic link to iron metabolism in pathogenic bacteria

Bacteria tightly regulate iron levels to prevent overload and the resulting oxidative stress. Accordingly, expression of genes involved in heme catabolism are often repressed by iron and activated by heme, sometimes in competing manners. Here we discuss several examples of metal-dependent transcriptional regulation, as well as heme regulation by a two-component system and a post-translational modification.

Many enzymes, including *isdG* and *isdI*, are regulated by the Ferric Uptake Regulator, or Fur (Carpenter et al., 2009; Troxell and Hassan, 2013). The *isdG* gene is part of a chromosomal locus that encodes a key heme uptake pathway and it is the terminal gene of the *isdCDEF/srtB/isdG* operon. This operon also encodes a membrane heme transporter (*isdCDEF*) and a dedicated heme import sortase (*srtB*) (Reniere and Skaar, 2008). Transcription of this operon is derived from the *isdC* promoter, divergently from the heme-receptor genes *isdA* and *isdB*. The *isdI* gene is the second gene in a bicistronic operon located elsewhere in the genome (Reniere and Skaar, 2008). The promoters controlling the expression of *isdG* and *isdI* each contain a Fur box. In the wild type strain, IsdG and IsdI levels increase when medium iron levels are decreased, however the enzymes levels remain stationary in a null *fur* mutant (Reniere and Skaar, 2008). This shows that expression is repressed in the presence of iron, although IsdG is also stabilized in the presence of heme (discussed below). Of note, *isdG* and *isdI* double knock-out mutants still exhibit growth with heme as the only iron source, suggesting *S. aureus* contains a third heme degrading mechanism (Reniere and Skaar, 2008).

Similarly to *S. aureus, P. aeruginosa* also encodes two HOs, PigA/HemO and BphO; however, only PigA/HemO is involved in iron metabolism. The *pigA/hemO* gene, which is part of a Fur regulated gene cluster, is required for efficient use of heme iron by *P. aeruginosa* and can complement the phenotype of *hemO* mutants in *N. meningitidis* (Ratliff et al., 2001). The second HO, BphO, catalyzes the production of biliverdin used for the assembling the photoreceptor phytochrome, BphP (Wegele et al., 2004). BphO is transcribed upstream from BphP in a bicistronic operon that is regulated by cell density and expressed independently of the iron status. PhuS, however, only transfers heme to PigA/HemO. In addition to studies demonstrating heme transfer from holo-PhuS to PigA/HemO, ^13^C-heme isotopic labeling studies showed that a Δ*phuS* mutant degrades externally provided heme using both of its HOs, PigA/HemO and BphO (Barker et al., 2012). Since the wild type strain uses only PigA/HemO to degrade externally provided heme, these experiments support the role of PhuS as intracellular heme chaperon that shuttles the heme specifically to PigA/HemO. Interestingly, these studies also implicated PhuS as a modulator of heme uptake since the mutant strain demonstrated altered expression of the heme uptake machinery.

The expression of the *chuZ* gene, an enzyme with a PNPOx-like domain found in *C. jejuni*, is also regulated by Fur. The *chuZ* gene, is located directly upstream of the *chuABCD* genes, which together code for a heme ABC transporter (Ridley et al., 2006). Mutants that lack either *chuZ* or *chuA* are unable to utilize heme as an iron source, but mutants that lack *chuB, chuC*, or *chuD* are only partly affected, suggesting an additional heme uptake system. Two fur boxes lie in between *chuZ* and *chuA*. Using transcriptional fusions and competitive EMSA assays, Ridley and colleagues demonstrated that both promoters are expressed in an iron-dependent manner and bind to Fur protein *in vitro* (Ridley et al., 2006).

The Diphtheria Toxin Repressor (DtxR) is mechanistically similar to Fur and controls the expression of genes involved in metal homeostasis and pathogenesis of many Gram-positive bacteria (Nobles and Maresso, 2011; Merchant and Spatafora, 2014). The transcription of *hmuO* from *C. diphtheriae* is repressed in the presence of iron by DtxR (and activated by heme, as will be described below) (Schmitt, 1997a). EMSA and DNase I footprinting experiments showed that DtxR binds in a metal-dependent manner in the promoter region of *hmuO* in *C. diphtheriae* (Schmitt, 1997a). The expression of *hmuO* in *C. ulcerans* appears to be controlled differently (Kunkle and Schmitt, 2007). Although the *hmuO* promoter in *C. ulcerans* contains a putative DtxR like box, promoter fusion studies revealed that it is only weakly repressed by iron. The basal expression of *hmuO* grown in the absence of heme is significantly higher in *C. ulcerans* than in *C. diphtheriae*, although the mechanism is unclear. A null *hmuO C. ulcerans* mutant cannot survive with heme as the only iron source while the phenotype of *C. diphtheriae* mutants vary, exhibiting moderate to no growth attenuation depending on the strain (Kunkle and Schmitt, 2007). Together these observations suggest that *hmuO* is important for the extraction of iron from heme in *C. ulcerans* and in some strains of *C. diphtheriae*, and that *C. diphtheriae* likely encodes additional factor(s) that allow the use of heme iron.

A DtxR-like protein named MtsR regulates the transcription of *hupZ*, a recently described enzyme in *S. pyogenes* (Bates et al., 2005; Sachla et al., 2016). MtsR binds to DNA in the presence of either iron or manganese. It was first recognized as the repressor of the *sia* operon (*shr/shp/siaABCDEFG*) which encodes a central heme import pathway and of the *mtsABC* metal transporter (Hanks et al., 2006; Sachla et al., 2016). Global transcription analysis of null *mtsR* mutants established MtsR as a global regulator that controls the expression of many *S. pyogenes* genes, including iron and heme uptake, metal homeostasis and important virulence factors (Toukoki et al., 2010). A two-gene cluster encoding a putative surface receptor and a cytoplasmic enzyme, later named HupZ, was identified in the MtsR regulon (Sachla et al., 2016). The *in vitro* activity of HupZ and its repression by MtsR implicate this enzyme in the utilization of heme as an iron source, but this role has yet to be established *in vivo*.

The expression of some heme associated genes is dependent on heme availability. The heme dependent-regulation of *hmuO* from *C. diphtheriae* is the first case where bacteria were shown to use two-component systems to modulate the expression of HOs. Out of the eleven two-component systems encoded by *C. diphtheriae*, two (ChrAS and HrrAS) modulate heme homeostasis. Both regulatory systems activate the *hmuO* promoter in the presence of heme, but the ChrAS system is the dominant one (Bibb et al., 2007; Bibb and Schmitt, 2010). ChrAS also activates the transcription of *hrtAB*, an ABC transporter that functions in heme detoxification. While ChrAS and HrtAB both repress *hemA*, a heme biosynthesis gene. Fluorescence staining experiments demonstrated that the sensor kinase, ChrS, autophosphorylates then phosphorylates ChrA, the response regulator (Burgos and Schmitt, 2012). A conserved aspartate residue in ChrA (Asp50) was implicated as the phosphorylation site and shown to be important for ChrA binding to *hmuO* promoter. DNase I footprint experiments identified two 10-bp segments (designated as S2ho and S3ho) that protected by ChrA and are conserved in other ChrA regulated promoters. S3ho partially overlaps the DtxR binding box, suggesting that ChrA and DtxR compete for binding during growth in the presence of heme and iron. Mutagenesis and promoter fusion experiments confirm the importance of the both S2ho and S3ho in the regulated expression of *hmuO* (Burgos and Schmitt, 2012).

Heme availability also regulates IsdG, but via a post-translational modification (Reniere and Skaar, 2008; Reniere et al., 2011). As was previously mentioned, IsdG is the terminal gene of the *isd* operon. While the entire operon is repressed by Fur in the presence of iron, only the concentration of IsdG is enhanced in the presence of heme. A pulse-chase and immunoprecipitation study of IsdG (expressed from a constitutive promoter) found that the half-life of IsdG increased 2.5 fold in medium containing heme, suggesting that heme stabilizes the protein (Reniere and Skaar, 2008). The stability of a catalytically inactive IsdG was similarly increased by heme, indicating that it is the heme and not the reaction product (staphylobilin) that facilitates stability. Analysis of a collection of chimeric proteins consisting of different IsdI and IsdG segments implicated an internal, β-strands containing, fragment in IsdG degradation. Further investigations identified a flexible loop near the heme-binding site that is required (but not sufficient) for proteolysis in the absence of heme (Reniere et al., 2011). It was hypothesized that the differential regulation of IsdG/I allows *S. aureus* to specifically adjust the expression of its HOs to the different host environment. Interestingly, while both enzymes are required for full pathogenesis, *isdG* mutants are more impaired in heart and kidney colonization models in comparison to *isdI* mutants (Reniere and Skaar, 2008).

## Future direction

Advances in the fields of biochemistry and bioinformatics are improving the understanding of bacterial heme degradation. There is, however, still more to learn about these enzymes, such as why MhuD retains an aldehyde while IsdG does not and whether proteins with HemS or PNPOx domains degrade heme or protect from oxidative stress *in vivo*. Additionally, several species of bacteria lack proteins with homology to reported heme degrading enzymes and several enzymes were discovered that do not use oxidation to open the heme ring. One such protein is the recently discovered ChuW, a radical S-adenosylmethionine methyltransferase from *E. coli* O157-H7, which appears to degrade heme use a primary carbon radical to initiate a methyl transfer instead of binding O_2_ (Lamattina et al., 2016). In *E. coli* K12 the enzymes YfeX and EfeB also liberate iron from heme without breaking the protoporphyrin ring (Létoffé et al., 2009).

More research is needed to understand the mechanism for many of these proteins and would allow better understanding of their cellular role. Oxidative heme degradation requires an electron supply in addition to molecular oxygen and the electron donor for most bacterial HOs are unknown. Mammalian HOs use CPR and in the absence of a known physiological electron donor, *in vitro* heme degradation is achieved using CPR or an alternative reducing agent (e.g., trolox or ascorbate). Often, different reducing agents produce different degradation kinetics and can halt the reaction at different stages, therefore they give a limited view of the *in vivo* reaction. In *P. aeruginosa*, the NADPH-dependent ferredoxin reductase, Fpr, was implicated as the physiological electron donor for PigA/HemO due to its ability to sustain catalytic activity during *in vitro* studies (Wang et al., 2007). The two likely reductase partners for the Staphylococcal HOs are IruO (a pyridine nucleotide-disulfide oxidoreductase) and NtrA (a nitroreductase) (Hannauer et al., 2015). Severe defects in heme usage are exhibited by an *iruO ntrA* double mutant (but not with either of the single gene mutants) implicated these enzymes in the process of heme catabolism. The search for native electron donors for bacterial HOs is important to develop the understanding of heme catalysis at the molecular level. New advances in our ability to monitor heme degradations as they are carried out in the cell are likely to enhance this important area of bacterial physiology.

Understanding the downstream effects of heme degradation products, such as biliverdin, staphylobilin, and CO is another area of research that could be expanded. In mammals, biliverdin is released from HO-1 and reduced to bilirubin by the action of biliverdin reductase. In bacteria, the destiny of the bilin produced during heme catalysis and its role in the cell physiology remains largely unknown. A recent mouse study showed that over expression of the *E. coli* ChuS leads to attenuation of the innate immune system (Maharshak et al., 2015). This immunomodulation was achieved by decreasing the expression of the pro-inflammatory cytokine, IL-12 p40, and increasing expression of the anti-inflammatory cytokine, IL-10. Researchers speculated that cross-talk between mammalian HO-1 and enteric bacteria HOs might be a key component of maintaining healthy intestinal microbiota, making it an important area for farther study.

Since heme iron is used by many pathogenic bacteria, targeting heme uptake and degradation provides attractive targets for therapeutic inhibition. A direct role between HOs and virulence was established in only a few cases. Mice challenged with mutant *S. aureus* that lacked either *isdG, isdI*, or both genes, showed decreased bacterial loads in heart and kidney tissues compared to WT indicating that they are necessary for full pathogeneses (Reniere and Skaar, 2008). Deleting the *hemO* gene in *Leptosria interorgans*, which is required for growth on hemoglobin iron, resulted in a 50% increase of survival rate of infected hamsters (Murray et al., 2009). A better understanding of HOs function and the mechanisms that facilitate heme degradation during infection may lay the ground for new therapeutic designs.

## Conclusion

In many pathogenic bacteria, heme catabolism is carried out by HOs similar to either HO-1 or IsdG. Other bacteria encode enzymes that bind and degrade heme *in vitro*. These proteins fall into two families based on structure: enzymes that contain HemS motif and those with PNPOx domains. Proteins from these families are regulated by iron, support bacterial growth on heme iron, and/or aid in tolerance of heme and oxidative stress. It seems possible that proteins from these families might have evolved to serve separate functions in different bacteria. Already, PhuS was shown *in vivo* to shuttle heme to the HO, PigA/HemO. While *in vitro* degradation and additional investigations link proteins from these families to iron/heme metabolism, more work is required to determine their physiological role. Genetic studies suggest that even in bacteria with recognized HOs there are additional (likely non-homologous) enzymes that aid in heme degradation. This redundancy emphasizes the importance of this mechanism for bacterial physiology. For 50 years, this area has challenged both researchers and technology. While much has been learned about how pathogenic bacteria obtain iron, the complexity and diverse strategies employed by the bacteria could easily fascinate researchers for many more years.

## Author contribution

KL generated the figures and the sequence comparisons, and wrote the manuscript; ZE critically revised the manuscript. Both authors have made substantial, direct and intellectual contribution to the work, and have approved it for publication.

### Conflict of interest statement

The authors declare that the research was conducted in the absence of any commercial or financial relationships that could be construed as a potential conflict of interest. The reviewer MV and handling Editor declared their shared affiliation.
